# Development of a Visuoperceptual Measure for Fiberoptic Endoscopic Evaluation of Swallowing (V-FEES) in Adults with Oropharyngeal Dysphagia: An International Delphi Study

**DOI:** 10.3390/jcm12123875

**Published:** 2023-06-06

**Authors:** Reinie Cordier, Renée Speyer, Susan Langmore, Deborah Denman, Katina Swan, Daniele Farneti

**Affiliations:** 1 Department of Social Work, Education and Community Wellbeing, Northumbria University, Newcastle upon Tyne NE7 7XA, UK; reinie.cordier@northumbria.ac.uk; 2 Curtin School of Allied Health, Faculty of Health Sciences, Curtin University, Perth 6102, Australia; katina.swan@postgrad.curtin.edu.au; 3 Department of Health & Rehabilitation Sciences, Faculty of Health Sciences, University of Cape Town, Cape Town 7935, South Africa; 4 Department Special Needs Education, University of Oslo, 0318 Oslo, Norway; 5 Department of Otorhinolaryngology and Head and Neck Surgery, Leiden University Medical Centre, 1233 Leiden, The Netherlands; 6 Department of Otolaryngology Head-Neck Surgery, Boston University School of Medicine, Boston, MA 02118, USA; langmore@bu.edu; 7 Department of Linguistics, Macquarie University, Sydney 2109, Australia; deborah.denman@mq.edu.au; 8 St John of God Midland Public and Private Hospitals, St John of God Health Care, Perth 6056, Australia; 9 Department of Allied Health, The School of Medical and Health Sciences, Edith Cowan University, Perth 6027, Australia; 10 Audiologic Phoniatric Service, Otorhinolaryngology Department, Infermi Hospital, AUSL Romagna, 47900 Rimini, Italy; daniele.farneti@auslromagna.it

**Keywords:** FEES, fiberoptic endoscopic evaluation of swallowing, deglutition, swallowing disorders, measure, instrument development, content validity, psychometrics

## Abstract

Visuoperceptual evaluation of fiberoptic endoscopic evaluation of swallowing (FEES) is a commonly used assessment in dysphagia or swallowing disorders. Currently, no international consensus exists regarding which visuoperceptual measures to use for the analysis of FEES recordings. Moreover, existing visuoperceptual FEES measures are limited by poor and incomplete psychometric data, identifying an urgent need for developing a visuoperceptual measure to interpret FEES recordings. Following the COSMIN group’s (COnsensus-based Standards for the selection of health Measurement INstruments) psychometric taxonomy and guidelines, this study aimed to establish the content validity of a new visuoperceptual FEES (V-FEES) measure in adults with oropharyngeal dysphagia. Using the Delphi technique, international consensus was achieved among dysphagia experts across 21 countries, resulting in a new prototype measure for V-FEES, comprising 30 items, 8 function testing items (i.e., specific tasks performed by patients while observing and rating items), and 36 unique operationalisations (i.e., defining items into measurable factors that could be measured empirically using visuoperceptual observation). This study supports good content validity for V-FEES, including participants’ feedback on the relevance, comprehensiveness, and comprehensibility of the included items. Future studies will continue the instrument development process and determine the remaining psychometric properties using both the classic test theory (CTT) and item response theory (IRT) models.

## 1. Introduction

Since its introduction in the 1980s, fiberoptic endoscopic evaluation of swallowing (FEES) has been an important instrumental assessment used to evaluate dysphagia or swallowing problems [1]. Several clinical protocols [2,3,4] and visuoperceptual assessments [5,6,7,8] have been published since the FEES was introduced to practice and research. Dysphagia or swallowing disorders are a frequent symptom of one or more underlying anatomical abnormalities or impairments and disorders in cognitive, sensory and motor acts involved in transporting food and liquids from the mouth to the stomach [9]. Dysphagia can lead to reduced efficiency and safety of swallowing, failure to maintain hydration and nutrition, risk of choking and aspiration leading to pulmonary complications, and reduced quality of life [9,10]. Dysphagia may refer to oropharyngeal disorders involving upper digestive tract problems and esophageal disorders involving lower digestive tract problems (or a combination of these). The prevalence of oropharyngeal dysphagia in the general population has been estimated to range between 2.3 and 16% [11]. Pooled prevalence estimates of oropharyngeal swallowing problems determined by meta-analyses, for example, are as high as 42% in post-stroke [12], 31.5% (95% CI 8.9–68.4%) in head and neck oncology [13], 36.9% (95% CI 30.7–43.6%) in Parkinson’s disease [14], 44.8% (95% CI 40.4–49.2%) in multiple sclerosis [15], 50.4% (95% CI 36.0–64.8%) in cerebral palsy [16], and (95% CI 26.7–95.0%) in dementia 72.4% [13].

Prevalence data may differ depending on which screen or assessment has been used, but in general, instrumental assessments (i.e., endoscopic and videoradiographic recordings of the swallowing process) are considered to be the most optimal evaluation methods to identify dysphagia, especially because both ‘gold standard’ assessments can diagnose aspiration (including silent aspiration) and other physiological problems in the pharyngeal phase [17]. However, no international consensus exists regarding which visuoperceptual measures to use to analyse these video recordings. Moreover, insufficient psychometric evidence has been identified from the literature to recommend any individual measure as valid and reliable to interpret swallowing recordings [18]. Consequently, implementing assessments with poor psychometric qualities will undermine evidence-based practice and research as current health status or intervention effects cannot be objectified if measures lack psychometric robustness [19,20].

The lack of robust psychometric visuoperceptual measures to evaluate the ‘gold standard’ instrumental recordings identifies an urgent need for instrument development. A recent Delphi study aimed to achieve international consensus on the visuoperceptual evaluation of videoradiographic recordings of swallowing (VideoFluoroscopic Swallowing Study or VFSS) as a starting point for instrument development [21], but there is no such study yet for the evaluation of FEES recordings. The ongoing discussion in the literature about which instrumental assessment to use shows advantages and disadvantages for both FEES and VFSS. Videoradiography is associated with radiation exposure, expensive resources (e.g., equipment and required personnel including a physician and allied health clinician), and limited availability to clinicians. Conversely, videoendoscopy requires no radiation, is less expensive, and is usually more accessible to healthcare providers, but it cannot assess the oral phase of swallowing and shows a brief period of white-out during the actual swallow act. Apart from the listed advantages and disadvantages, the choice of implementing either FEES or VFSS in dysphagia care will also depend on the main purposes of the examination, as well as factors related to the clinical environment (such as availability and/or affordability).

The COnsensus-based Standards for the selection of health Measurement INstruments (COSMIN) group established an international consensus-based taxonomy, terminology, and definitions of measurement properties [22,23]. The framework comprises nine measurement properties within three domains: reliability, validity and responsiveness. In line with the COSMIN framework, content validity is the most important measurement property, referring to the degree to which an instrument’s content adequately reflects the construct to be measured [22]. A measure is considered of questionable value for either research or clinical practice if content validity is flawed or lacking. To meet the COSMIN criteria for good content validity, both recent literature on the construct of interest and clinical experts should be involved in the process of developing new measures. According to COSMIN, professionals should be asked about the relevance (all items should be relevant to the construct of interest within a specific population and context of use), comprehensiveness (no key aspects of the construct should be missing), and comprehensibility (the items should be understood by the target populations as intended) of the items of a measure [22].

Informed by the COSMIN guidelines [22], this study aims to report on the first step towards developing a visuoperceptual measure to evaluate endoscopic recordings of swallowing in oropharyngeal dysphagia. To ensure good content validity, this study reports on an international Delphi study involving dysphagia experts to seek agreement on definitions and items of a prototype of visuoperceptual measurement in FEES recordings.

## 2. Methods

### 2.1. Study Design

This study used the Delphi technique to develop group consensus between experts on a defined topic using a series of survey rounds as part of a structured process [24]. Consecutive Delphi surveys are modified based on the percentage of agreement and participants’ feedback from preceding survey rounds. Participants remain anonymous throughout all Delphi rounds to avoid bias and discourage individuals from dominating the consensus process. The same participants are invited to complete each Delphi round, although some participants may choose to withdraw during the Delphi process. Delphi rounds continue until group consensus has been reached or it becomes apparent that consensus cannot be reached. This study used online surveys (e-Delphi) to seek expert consensus regarding the items to be included in a visuoperceptual measure to evaluate FEES recordings in adult patients with oropharyngeal dysphagia.

### 2.2. Participants

To be eligible to participate in the Delphi study, participants needed to: (1) have English reading skills adequate for work (e.g., understanding of the main points of texts and technical terms within the participant’s field of expertise); and (2) have five (full-time equivalent) or more years clinical, research or teaching experience in dysphagia with a caseload of at least 50% or more related to adults with dysphagia (e.g., provision of clinical services, research, staff development, academic teaching) and using FEES.

### 2.3. Procedure

#### 2.3.1. Recruitment

This study was approved by the Curtin Human Research Ethics Committee (Curtin University, Perth, Australia: HRE2021-0187). Delphi participants were recruited via professional organisations (e.g., European Society for Swallowing Disorders), from the professional networks of the authors, through reviewing relevant publications in FEES, and by asking recruited participants to identify other potential participants (snowballing). Identified participants were sent an invitation and an information sheet about the Delphi study. Participants who accepted the invitation were sent a link to the first online Delphi survey. As all survey data were processed anonymously, all participants were reinvited to consecutive Delphi rounds regardless of whether they had completed previous rounds.

#### 2.3.2. e-Delphi Surveys

Definitions for main concepts and a list of potential measure items were constructed based on: (a) existing visuoperceptual measures in FEES as identified in a previous systematic review [18], (b) selected consensus-based definitions related to the visuoperceptual analysis of videoradiographic recordings of swallowing in oropharyngeal dysphagia as reported in a recent Delphi study [21], (c) other relevant international literature, and (d) group discussions between the authors. Potential items were presented to participants across three rounds via an online survey platform Qualtrics over fourteen months (December 2021–January 2023). Participants indicated consensus on definitions, comprehensibility and relevance using a five-point scale. In both the second and third Delphi rounds, participants were also asked about their choice of function testing (i.e., specific tasks performed by patients while observing and rating items) and the operationalisation thereof (i.e., defining items into measurable factors that could be measured empirically using visual perceptual observation). Participants who disagreed were asked to provide further details and suggestions for revision in open text boxes. In addition, participants were asked about the comprehensiveness of the preliminary measure and to identify missing items to capture underlying constructs fully. At the end of each Delphi survey, open-ended comment sections were available. Between Delphi rounds, participants received summarised findings on participants’ characteristics, percentage agreement on definitions and items, and revisions made using their feedback (i.e., rewording definitions, revising, and adding or deleting items). Samples of all three Delphi rounds are presented in Supplementary File S1, providing further details on structure and content.

### 2.4. Analysis

Survey responses were analysed using the Statistical Package for the Social Sciences [25]. Criteria for agreed consensus were defined before the first Delphi survey using a five-point scale (i.e., strongly disagree, disagree, neither agree nor disagree, agree, strongly agree). Consensus between participants was achieved if at least 70% of respondents indicated ‘Strongly agree’ or ‘Agree’ for the formulation of definitions, ease of understanding items (comprehensibility), the importance of including items (relevance), operationalisation of items, and function testing [26,27]. Participants’ responses to open-ended questions were analysed per item before rewording the original items or creating a new item based on participants’ suggestions. As proposed by participants, responses to open-ended questions on the measure’s comprehensiveness were grouped into themes where potential new items were identified based on the aggregated feedback. Overall, measure revisions were based on themes noted in most participants’ comments, feedback supported by literature, and identified gaps or ambiguities in items. The total number of Delphi rounds was to be determined by the level of agreement following each round. When performing data analysis, the authors were blinded to the identity of the participants.

## 3. Results

### 3.1. Delphi Participants

Potential participants were identified through professional organisations, authors’ networks, key publications and snowballing. The number of participants who agreed to participate in Delphi round one was 64, 52 for round two (response rate 81.3% [52/64], and 41 for round three (response rate 64.1% [41/64]. Table 1 presents the participants’ demographics.

Across Delphi rounds, the majority of participants were speech and language pathologists (71.9–78.0%), with the remaining participants being othorhinolaryngologists/phoniatricians (17.1–21.9%) or having been trained in another medical specialism such as neurology, pulmonology, rehabilitation or geriatrics (4.9–7.6%). Most participants had completed a higher degree by research (PhDs [56.1–63.5%] or Master’s degrees by research [28.8–37.5%]), and the other participants had completed Bachelor’s degrees (6.3–7.7%). On average, the number of years of working experience with FEES in adults with dysphagia varied among participants: 21.0% reported 5 to 10 years, 50.8% between 11 and 20 years, and 28.8% over 21 years of experience. The vast majority of participants worked in hospital settings (65.9–75.0%), working with patients with non-degenerative diseases/acquired neurological trauma (28.8–36.6%), oncology patients (24.4–26.9%), and patients with degenerative neurological disorders (13.5–14.6%) as the most frequent primary patient populations. Participants were spread across 21 countries and 5 continents: Asia (5.8–12.5%), Europe (43.9–48.1%), North America (26.6–29.3%), Oceania (9.4–17.1%) and South America (2.4–4.7%). Further details on participants’ demographics can be found in Table 1.

### 3.2. Delphi Process

In addition to the structure and content of Delphi rounds with examples (Supplementary File S1), a summarised overview of the Delphi process is outlined in Table 2.

#### 3.2.1. Delphi Round I

The first Delphi round included 32 items and definitions related to the visuoperceptual analysis of video recordings of swallowing in oropharyngeal dysphagia. A total of 21 items originated from a previous Delphi study on instrument development in videofluoroscopy of swallowing by Swan et al. (2021) [21]. An additional 11 items were defined based on other relevant literature and authors’ expert opinions. As the first 21 items had achieved international consensus on definition and comprehensibility in the previous Delphi study by Swan et al. (2021) [21], participants were only asked to rate the relevance of these 21 items for the visuoperceptual evaluation of FEES recordings using a five-point ordinal scale (strongly disagree to strongly agree). For the remaining 11 items, participants rated both the relevance and the degree of agreement with each item’s suggested definitions and comprehensiveness using similar ordinal scales. If participants disagreed or strongly disagreed, they were invited to comment in an open text box.

Fifteen items were included without requiring revisions, whereas seven were slightly reworded. The following six items were excluded as they were not considered relevant for FEES evaluation: ‘Arytenoid tilting’, ‘Base of tongue to posterior pharyngeal wall approximation’, ‘Epiglottic return to rest position’, ‘Linguavelar seal’, ‘Tongue base activity’, and ‘Swallow initiation’. The relevance percentage scores for these six items ranged between 39.1% and 65.8%. The original item ‘Piecemeal deglutition’ and its corresponding definition were also deleted, but the item name was used to rename ‘Clearing swallow (oral)’. ‘Nasopharynx penetration’ was the eighth item that was deleted as participants doubted its visibility in FEES. Two items (‘Premature spillage [Liquids]’ and ‘Premature spillage [Other than liquids]’) moved to the second Delphi round after renaming both item names (replacing ‘Premature’ with ‘Posterior’) and rewording their definitions. Further details can be found in Table 2 (Overview of the Delphi process) and Table 3 (Relevance of items to the visuoperceptual evaluation of FEES).

#### 3.2.2. Delphi Round II

The second Delphi round consisted of three sections. The first section included the two items that were carried over from the first round (‘Posterior spillage [Liquids]’ and ‘Posterior spillage [Other than liquids]’). Both items were accepted without further rewording. The second section targeted the comprehensibility of the measure under development. Participants were asked to study a list of all included items from the previous Delphi round (*n* = 24, including both items from the first section) and identify any missing items relevant for visuoperceptual evaluation in FEES. Four new items were suggested (‘Esophageal backflow’, ‘Symmetry’, ‘Pooling of secretions’, and ‘Respiratory rate and effort’). Based on participants’ feedback, all four items and corresponding definitions were carried over to the third Delphi round for agreement ratings.

The final section focused on how to assess each item or aspect of the item (i.e., operationalisation) and, where applicable, the tasks that needed to be performed by patients (i.e., function testing) while observing and rating the item. For each operationalisation, examples of possible response scales were provided. Several operationalisations and function testing items were listed for participants’ evaluation. For example, for the item ‘Aspiration’, the following three aspects of the item were presented (each with a different operationalisation): (1) ‘Aspiration of material (e.g., liquids, solids)’ operationalised by ‘Volume of aspirated bolus (e.g., nil material, a small amount of material, a large amount of material)’; (2) ‘Patient response to aspiration (i.e., an overt sign of aspiration, such as cough/throat-clear)’ operationalised by ‘Cough (e.g., immediate cough, late cough, no response)’; and, (3) ‘Success of ejecting aspirated bolus’ operationalised by ‘Success in ejecting material from the airway (e.g., complete clearing, incomplete clearing, nil clearing)’. For the item ‘Pharyngeal constriction’, both a ‘Saliva swallow’ and a ‘High pitched strained ‘eeee’ (pharyngeal squeeze manoeuvre)’ were suggested to test function. Participants rated their level of agreement with operationalisations and function testing using the same five-point ordinal scales as in previous Delphi sections, including the option for comments in case of disagreement (see Supplementary File S1).

Agreement ratings for function testing (*n* = 10) and operationalisations (*n* = 35) for all 24 items were determined by considering the comments listed by participants. This resulted in a total of eight function testing items and thirty-nine operationalisations being included in the third Delphi round. Similarly, based on participants’ feedback, two items (‘Vocal fold medialisation’ and ‘Laryngeal vestibule closure’) were rephrased again and included in the third Delphi round.

#### 3.2.3. Delphi Round III

The third Delphi round consisted of three sections. The first section asked participants to rate their agreement with both revised and renamed items from the second round. One revised item was accepted without need for rewording. However, even though the other item, ‘Laryngeal vestibule closure’, was considered relevant to the visuoperceptual evaluation of FEES, participants could still not agree on a definition after three Delphi rounds and thus it was excluded. The second section asked about the relevance and agreement on definitions for all four new items, as suggested in the second Delphi round. Participants agreed on including three new items, but the fourth item, ‘Respiratory rate and effort’, was excluded due to low relevance ratings. Further, using participants’ feedback, the items ‘Regurgitation’ and ‘Esophagopharyngeal backflow’ were combined into a new item and renamed ‘Esophageal backflow’.

The third section of this final Delphi round asked about participants’ agreement with the scales used to operationalise the included items using the same five-point ordinal scales as used in previous rounds. For example, after participants agreed in round two to operationalise the item ‘Bolus holding (to command)’ by the presence of material in the pharynx, a three-point ordinal scale (i.e., Nil material present in the pharynx; <one-third of bolus present in pharynx; ≥one-third of bolus present in pharynx) was presented for agreement ratings in round three. In the end, 29 out of 39 operationalisations presented were accepted for inclusion in the prototype measure. Table 4 provides an overview of agreement ratings of function testing and operationalisations.

#### 3.2.4. Final Prototype

This Delphi study resulted in the prototype measure, Visuoperceptual measure for Fiberoptic Endoscopic Evaluation of Swallowing (V-FEES). The final prototype measure comprises 30 items, 8 function testing items and 36 unique operationalisations (see Table 4). The final percentage agreement for the relevance of the included items to visuoperceptual evaluation to FEES is presented in Table 3: mean 86.7% (SD 9.85%). Table 5 provides an overview of the included items and percentage agreement with definitions (mean 84.0%; SD 6.71%), and Table 4 shows participants’ agreement with function testing (mean 81.5%; SD 8.60%) and final operationalisations (mean 75.8%; SD 10.21%).

## 4. Discussion

### 4.1. Content Validity

This study is the first step towards developing and validating a visuoperceptual measure to evaluate fiberoptic endoscopic recordings of swallowing in adults with oropharyngeal dysphagia. To meet the COSMIN guidelines for content validity [23], an international Delphi study was conducted to seek agreement among dysphagia experts on definitions and items of a prototype measure and achieve consensus on function testing and operationalisations for the included items, covering all three aspects of content validity (i.e., relevance, comprehensibility and comprehensiveness).

An initial number of 64 dysphagia experts completed the first Delphi round, of whom 41 experts also completed the final, third Delphi round. As the COSMIN guidelines consider a minimum of 30 experts to support adequate and a minimum of 50 experts to support very good methodological quality for quantitative studies (e.g., Delphi study) [22], the current study meets the COSMIN standards for the required number of respondents. Further, most participants had completed a higher degree by research (92.3–93.7%), and most experts reported having over ten years of experience working with FEES in adult patients with dysphagia (73.4–82.9%) of whom between a quarter and a third of experts (23.4–34.6%) noted over 20 years of experience. Therefore, the Delphi participants represented a highly qualified and experienced dysphagia expert group.

### 4.2. Instrument Development: V-FEES

#### 4.2.1. Definitions and Items

The final prototype measure V-FEES includes 12 item definitions from a previously published international Delphi study [21], whereas consensus agreement was achieved for the remaining new definitions in the current study. Overall, relevance ratings were high (mean 86.7%), with seven items (i.e., ‘Aspiration’, ‘Cough (reflexive)’, ‘Penetration’, ‘Pharyngeal residue’, ‘Pooling of secretions’, ‘Premature spillage [Liquids]’, and ‘Silent aspiration’) showing ratings above 95% (range 96.9–100%). Similarly, agreement of item definitions was high (mean 84%), with one item (‘Esophageal backflow’) achieving ratings above 95% (97.2%) after three Delphi rounds. For one item (‘Laryngeal vestibule closure’), however, participants could not agree on a definition despite high relevance ratings (78.1%), after which the item was excluded from the prototype. For all other items, disagreements about terminology and phrasing were resolved within three Delphi rounds.

#### 4.2.2. Function Testing and Operationalisations

Although participants agreed on function testing with minimal need for discussion, achieving consensus on the scales used to operationalise the included items was more challenging. Two recurrent topics for discussion remained unresolved. The first point of contention involved describing the location of residue or material in the pharynx. Participants disagreed on anatomical boundaries and reference scalars. As a compromise, the authors agreed that anatomical descriptors would be augmented by providing example pictures for each level of the location scales. The second point of contention involved how to report on volumes of material or bolus. The authors decided, based on participants’ feedback, to retain three-point ordinal scales as suggested (e.g., item ‘Pharyngeal residue’: (1) no residue or minimal coating [none–trace)]; (2) <one-third of bolus present in pharynx [mild–moderate]; (3) ≥one-third of bolus present in pharynx [severe]). During the next stage of instrument development, the implementability and reliability of volume scales will be evaluated.

### 4.3. Strengths and Limitations

An important strength of the current Delphi study is that it did not solely focus on participants’ percentage agreement with definitions, items, function testing and operationalisations but emphasised incorporating opportunities for feedback and discussion. Participants were encouraged to provide the logic for their ratings and comment on the study in general after each Delphi round. The authors used these arguments and comments to make decisions about the wording of items and the conceptualisation of response options. In addition, participants were informed about the previous round’s overall results between Delphi rounds, including revisions made based on experts’ feedback. This approach ensured that participants’ views and opinions were carefully considered in constructing the V-FEES.

However, even though this Delphi study represented many different geographical locations (i.e., 21 countries across 5 continents) and experts from various professional backgrounds, study outcomes were strongly influenced by the viewpoints of the included participants. Furthermore, participant dropout across Delphi rounds may impact results [24], even though the completion rate is considered to be within the expected rate for web-based survey studies [28,29]. Finally, this Delphi study is the first step towards developing the V-FEES. The current results do not address whether the items are valid or can be measured reliably. Future research will focus on determining the psychometric properties of the V-FEES.

### 4.4. Future Research

Achieving consensus among international experts on definitions and items of a prototype visuoperceptual measure for FEES recordings (V-FEES) supports the content validity of the newly developed measure. This constitutes an important milestone as content validity is considered a measure’s most important psychometric property according to the COSMIN framework [22]. Future studies will trial the V-FEES in patients with dysphagia to determine its psychometric properties using both classic test theory (CTT) and item response theory (IRT; Rasch analyses).

The COSMIN framework will guide the psychometric evaluation of V-FEES. The internal structure of V-FEES will be defined by evaluating structural validity and internal consistency, after which reliability, measurement error, measurement invariance, and hypotheses testing for construct validity (e.g., convergent validity) will be assessed. Responsiveness will be evaluated by comparing pre- and post-treatment data and reporting on the measure’s sensitivity to change. Because no internationally agreed ‘gold standard’ in visuoperceptual evaluation of FEES is available, criterion validity cannot be determined. Lastly, although not considered psychometric properties, the feasibility and interpretability of V-FEES in daily clinical practice will be evaluated by assigning qualitative meaning to quantitative scores. Following the COSMIN guidelines and using robust psychometric methodologies, V-FEES aims to be the first valid and reliable visuoperceptual measure for FEES based on international expert consensus.

## 5. Conclusions

This study has reported on the first steps towards validating a visuoperceptual measure to evaluate FEES recordings of swallowing in adults with oropharyngeal dysphagia by establishing the content validity of the V-FEES. Following COSMIN guidelines, an international Delphi study among dysphagia experts resulted in a new prototype measure comprising 30 items, 8 function testing items and 36 unique operationalisations. The findings from the current study support good content validity by incorporating participants’ feedback on the relevance, comprehensiveness, and comprehensibility of included items. Following the instrument development process, future studies will determine the psychometric properties of V-FEES using both classic test theory (CTT) and item response theory (IRT).

## Figures and Tables

**Table 1:** Participants’ demographics.

	ROUND ONE	ROUND TWO	ROUND THREE
**Number of participants**	**N = 64**	**N = 52**	**N = 41**
**Demographics**	**Frequency (%)**	**Frequency(%)**	**Frequency(%)**
**Continent of residence**			
Asia (Participants; Countries)	8 (12.5%; 4) (India [*n* = 1], Israel [*n* = 2], Japan [*n* = 4], Turkey [*n* = 1])	3 (5.8%; 3) (India, Israel, Turkey)	3 (7.3%; 2) (Israel, Turkey)
Europe (Participants; Countries)	30 (46.9%; 10 ^a^)	25 (48.1%; 9 ^b^)	18 (43.9%; 8 ^c^)
North America (Participants; Countries)	17 (26.6%; 2) (Canada [*n* = 2], United States [*n* = 15])	15 (28.8%; 2) (Canada [*n* = 1], United States [*n* = 14])	12 (29.3%; 2) (Canada [*n* = 1], United States [*n* = 11])
Oceania (Participants; Countries)	6 (9.4%; 2) (Australia [*n* = 4], New Zealand [*n* = 2])	7 (13.5%; 2) (Australia [*n* = 4], New Zealand [*n* = 3])	7 (17.1%; 2) (Australia [*n* = 4], New Zealand [*n* = 3])
South America (Participants; Countries)	3 (4.7%; 2) (Argentina [*n* = 2], Colombia [*n* = 1])	2 (3.8%; 2) (Argentina, Colombia)	1 (2.4%; 1) (Argentina)
**Highest qualification *(related to work in the field of dysphagia)***			
Bachelor	4 (6.3%)	4 (7.7%)	3 (7.3%)
Master	24 (37.5%)	15 (28.8%)	15 (36.6%)
PhD	36 (56.3%)	33 (63.5%)	23 (56.1%)
**Profession**			
Speech Language Pathologist	46 (71.9%)	40 (76.9%)	32 (78.0%)
(Otorhino)laryngologist/Phoniatrician	14 (21.9%)	8 (15.4%)	7 (17.1%)
Other Medical Specialist	4 (6.2%)	4 (7.6%)	2 (4.9%)
**Primary role**			
Clinician	38 (59.4%)	32 (61.5%)	28 (68.3%)
Clinical supervisor	8 (12.5%)	5 (9.6%)	6 (14.6%)
Researcher	11 (17.2%)	9 (17.3%)	6 (14.6%)
Academic	7 (10.9%)	6 (11.5%)	1 (2.4%)
**Practice setting (Primary)**			
Hospital	48 (75.0%)	39 (75.0%)	27 (65.9%)
Private practice	0 (0%)	3 (5.8%)	0 (0%)
Residential aged/Disability care	2 (3.1%)	3 (5.8%)	3 (7.3%)
Community health centre	4 (6.3%)	1 (1.9%)	2 (4.9%)
University/Education sector	8 (12.5%)	4 (7.7%)	8 (19.5%)
Research/Student	2 (3.1%)	2 (3.8%)	1 (2.4%)
**Practice setting (secondary)**			
Hospital	7 (10.9%)	3 (5.8%)	9 (22.0%)
Private practice	7 (10.9%)	N.A.	3 (7.3%)
Residential aged/Disability care	0 (0%)	20 (38.5%)	1 (2.4%)
Community health centre	2 (3.1%)	4 (7.7%)	4 (9.8%)
University/Education sector	23 (35.9%)	13 (25.0%)	11 (26.8%)
Research/Student	5 (7.8%)	2 (3.8%)	1 (2.4%)
No secondary practice setting	20 (31.3%)	10 (19.2%)	12 (29.3%)
**Patient populations (Primary)**			
Non-degenerative/Acquired neurological trauma	19 (29.7%)	15 (28.8%)	15 (36.6%)
Degenerative neurological disorders	9 (14.1%)	7 (13.5%)	6 (14.6%)
Oncology	17 (26.6%)	14 (26.9%)	10 24.4%)
Geriatrics	3 (4.7%)	3 (5.8%)	2 (4.9%)
Gastroenterology	1 (1.6%)	3 (5.8%)	1 (2.4%)
Respiratory diseases	1 (1.6%)	2 1.9%)	1(2.4%)
Other	8 (12.5)	5 (9.6%)	5 (12.2%)
**Patient populations (Secondary)**			
Non-degenerative/Acquired neurological trauma	13 (22.4%)	14 (26.9%)	5 (12.2%)
Degenerative neurological disorders	14 (21.9%)	8 (15.4%)	8 (19.5%)
Oncology	8 (12.5%)	7 (13.5%)	8 (19.5%)
Geriatrics	4 (6.3%)	8 (15.4%)	2 (4.9%)
Gastroenterology	2 (3.1%)	0	3 (7.3%)
Respiratory diseases	6 (9.4%)	3 (5.8%)	4 (9.8%)
Other	6 (9.4%)	3 (5.8%)	4 (9.8%)
No secondary patient population	5 (7.8%)	4 (7.7%)	6 (14.6%)
**Years of experience**			
5–10 yrs	17 (26.6%)	10 (19.2%)	7 (17.1%)
11–15 yrs	14 (21.9%)	11 (21.2%)	13 (31.7%)
16–20 yrs	18 (28.1%)	13 (25.0%)	10 (24.4%)
21–30 yrs	13 (20.3%)	15 (28.8%)	9 (22.0%)
>30 yrs	2 (3.1%)	3 (5.8%)	2 (4.9%)

^a^ Austria [*n* = 2], Croatia [*n* = 2], Finland [*n* = 3], France [*n* = 2], Germany [*n* = 7], Greece [*n* = 1], Slowakia [*n* = 2], Sweden [*n* = 2], the Netherlands [*n* = 1], United Kingdom [*n* = 8]. ^b^ Austria [*n* = 3], Finland [*n* = 2], France [*n* = 1], Germany [*n* = 4], Greece [*n* = 1], Italy [*n* = 1], Sweden [*n* = 2], the Netherlands [*n* = 1], United Kingdom [*n* = 10]. ^c^ Austria [*n* = 2], Finland [*n* = 2], Germany [*n* = 2], Greece [*n* = 1], Italy [*n* = 1], Sweden [*n* = 2], the Netherlands [*n* = 1], United Kingdom [*n* = 7].

**Table 2:** Overview of Delphi process.

Round (N_participants_)	Content	Results
**Round I** **(N = 64)**	**SECTION I: Items and definitions** (*n* = 21; Swan et al., 2021 [21]) **Questions ^ab^ (5-point ordinal scale)** This item is important to include (Relevance)	**ITEMS & DEFINITIONS** **Include ^c^** Agreement: no changes required (*n* = 15) Agreement: minimal rewording ^d^ (*n* = 7) **Exclude** Lack of agreement (*n* = 7) Not observable in FEES ^d^ *(n* = 1) **Include Round II** Reworded ^d^ (*n* = 2)
	**SECTION II: Items and definitions** (*n* = 11) **Questions ^b^ (5-point ordinal scale)** This item is important to include (Relevance) Rate your level of agreement with the definition This item is easy to understand (Comprehensibility)	
**Round II** **(N = 52)**	**SECTION I: Reworded items and definitions from Round I** (*n* = 2) **Questions ^b^ (5-point ordinal scale)** This item is important to include (Relevance) Rate your level of agreement with the definition This item is easy to understand (Comprehensibility)	**ITEMS & DEFINITIONS** **Include ^c^** Agreement (reworded items from Round I): no changes required (*n* = 2) **Include Round III** Reworded ^d^ (*n* = 2) New items: comprehensiveness (*n* = 4) **FUNCTION TESTING** **Include ^c^** Agreement: no changes required (*n* = 8) **Exclude** Redundancy (lower agreement of two function testing for the same item): (*n* = 2) **OPERATIONALISATION** **Include** (*n* = 25) **Exclude** (*n* = 10)
	**SECTION II: Comprehensiveness** List of all included items from Delphi Round I. **(Open) Question** Focus on comprehensiveness; are any observable items missing?	
	**SECTION III: Operationalisations** (*n* = 35) and **Function testing** (*n* = 10) **Questions ^b^ (5-point ordinal scale)** *If applicable*: Rate your level of agreement with the patient task (function testing) Rate your level of agreement with the operationalisation	
**Round III** **(N = 41)**	**SECTION I: Reworded items and definitions from Round II** (*n* = 2) Rate your level of agreement with the definition This item is easy to understand (Comprehensibility)	**ITEMS & DEFINITIONS** **Include ^c^** Agreement (reworded item from Round II): no changes required (*n* = 1) Agreement (new items): no changes required (*n* = 3) **Exclude** Lack of agreement reworded item Round II (*n* = 1) Lack of agreement on new items (*n* = 1) Overlap between 3 items: 2 items Round I replaced by one new item (*n* = 2) **OPERATIONALISATION (SCALES)** **Include** (*n* = 29) **Exclude** (*n* = 10)
	**SECTION II: New items and definitions from Round II** (*n* = 4) **Questions ^b^ (5-point ordinal scale)** This item is important to include (Relevance) Rate your level of agreement with the definition This item is easy to understand (Comprehensibility)	
	**SECTION III: Operationalisations (scales)** (*n* = 39) **Questions ^b^ (5-point ordinal scale)** Rate your level of agreement with the operationalisation	

^a^ Items from Swan et al. (2020) [21] were rated for relevance for FEES only as items resulted from a previous Delphi study on visuoperceptual evaluation of videofluoroscopic evaluation of swallowing recordings, having achieved international consensus on definition and comprehensibility. ^b^ If ‘Disagree’ or ‘Strongly disagree’, changes can be suggested in comment boxes. ^c^ Consensus agreement is defined as ≥70% of participants rating ‘Strongly agree’ or ‘Agree’. ^d^ Participants’ feedback on definition and comprehensibility.

**Table 3:** Relevance of items to visuoperceptual evaluation of FEES.

Item ^a^ (Alphabetical Order)	Renamed ^b^	% Agreement	Delphi Round
Arytenoid medialisation	***Vocal fold medialisation***	***75.0***	I
Arytenoid tilting		57.8	I
***Aspiration***		***98.5***	I
Base of tongue to posterior pharyngeal wall approximation		64.1	I
***Bolus holding (to command)***		***81.3***	I
Clearing swallow (oral)	***Piecemeal deglutition*** ^c^	***78.1***	I
***Clearing swallow (pharyngeal)***		***93.7***	I
***Cough (reflexive)***		***96.9***	I
***Cough (voluntary)***		***92.2***	I
Dry swallow	***Saliva swallow***	***87.5***	I
***Epiglottic retroflexion***		65.6 ^d^	I
Epiglottic return to rest position		62.5	I
***Esophageal backflow* ^e^**		***85.3***	III
Laryngeal vestibule closure		***78.1*** ^f^	I
Laryngopharyngeal backflow ^e^	Esophagopharyngeal backflow ^e^	***92.2*** ^e^	I
Linguavelar seal		39.1	I
Nasopharynx penetration		***73.5*** ^g^	I
***Penetration***		***96.8***	I
Pharyngeal contraction	***Pharyngeal constriction***	***87.5***	I
***Pharyngeal residue***		***98.5***	I
Piecemeal deglutition ^c^		***71.8*** ^c^	I
***Pooling of secretions***		***100***	III
Premature spillage [Liquids]	***Posterior spillage [Liquids]***	***98.1***	II
Premature spillage [Other than liquids]	***Posterior spillage [Other than liquids]***	***84.6***	II
Regurgitaton ^e^		***93.7*** ^e^	I
Respiratory rate and effort		61.0	III
***Sensory testing***		***76.5***	I
***Silent aspiration***		***96.9***	I
Spontaneous swallow	***Spontaneous saliva swallow***	***78.2***	I
Swallow initiation		65.8	I
***Swallow reaction time***		***75.0***	I
***Symmetry***		***90.3***	III
Tongue base activity		62.5	I
***Tracheal residue***		***90.6***	I
***Velopharyngeal closure***		***79.7***	I
***White out***		***73.5***	I

Note. Percentage agreement values are in bold and italic if they exceeded the minimum % agreement of 70%; Item names in bold are included in prototype measure. ^a^ Original name Delphi round I. ^b^ Renamed based on participants’ feedback. ^c^ Original item/definition ‘Piecemeal deglutition’ (Round I) deleted. Original item ‘Clearing swallow (oral)’ renamed ‘Piecemeal deglutition’ (Round II). ^d^ Participants’ discussion on the visibility of items. Authors’ decision to include rating option ‘not observed’ in prototype measure. ^e^ Domains ‘Regurgitation ‘ and ‘Esophagopharyngeal backflow’ replaced by ‘Esophageal backflow’ based on participants’ feedback and authors’ discussion. ^f^ Ongoing participants’ discussion on definition during all Delphi rounds. Round I: Agreement with definition 70.1%; Round II: Ongoing participants’ discussion (open text box); Round III: Agreement with revised definition 70.8%. Authors’ decision to exclude as ongoing marginal agreement on the definition. ^g^ Participants’ feedback (Round I): not observable in FEES.

**Table 4:** Agreement function testing and operationalisations (prototype measure).

Item (*Alphabetical Order*)	Function Testing (*If Applicable*)	% Agreement	Operationalisation	% Scale Agreement ^a^
*Aspiration*: *Aspiration* of material (e.g., liquids, solids)			I. Volume of aspirated bolus ^b^	63.4%
			II. Timing of spiration	90.3%
*Aspiration*: Patient response to aspiration (i.e., overt sign of aspiration, such as cough/throat-clear)			Cough or throat clearing	85.3%
*Aspiration*: Success of ejecting aspirated bolus			Success in ejecting material from the airway	82.9%
*Aspiration*: Cough on demand			Success in ejecting material from the airway	87.8%
Bolus holding (to command)	Hold bolus at least for five seconds	88.5%	I. Presence of material in pharynx ^b^	61.0%
			II. Location of material in pharynx ^c^	61.0%
Clearing swallow (pharyngeal)			Presence of clearing swallow and success of clearing.	80.5%
Cough (reflexive)			*As per cough (Voluntary)*	--
Cough (voluntary)	Cough on command (no bolus)	98.1%	Success in arytenoid adduction/closing vocal folds, followed by the brisk opening of the tightly closed larynx	80.5%
Epiglottic retroflexion			Visualisation of the epiglottis in retroflexed position immediately after white-out ^d^	73.1%
Esophageal backflow			Presence of material returned from the esophagus	85.4%
*Penetration*: *Penetration* of material (e.g., liquids, solids)			I. Volume of penetrated bolus ^b^	65.9%
			II. Depth of penetrated bolus ^c^	87.8%
			III. Timing of penetration	90.3%
*Penetration*: Patient response to penetration (i.e., an overt sign of penetration, such as cough/throat-clear/swallow) and success in clearing			Patient response and success in clearing	75.6%
Pharyngeal constriction	Saliva swallow or add a sip of water (1 mL) ^d^	76.9%	Medialisation of the lateral pharyngeal walls during swallowing	82.9%
Pharyngeal residue			I. The amount of material present in pharynx ^b^	65.9%
			II. The location of material present in pharynx ^c^	65.9%
Piecemeal deglutition			Number of portions bolus is divided into	75.6%
*Pooling of secretions*: *Pooling of secretions* in pharynx			I. The volume of secretions present in pharynx ^b^	70.7%
			II. The location of secretions present in pharynx ^c^	80.5%
			III. Appearance of secretions ^d^	51.2%
*Pooling of secretion*: Patient response to pooling of secretions in pharynx (e.g., cough, throat-clear or swallow) and success of ejecting secretions from the pharynx			Patient response and success in clearing	78.0%
*Pooling of secretion*: Clearing on command (e.g., cough, swallow)			Success in ejecting material from the pharynx	78.1%
Posterior spillage [Liquids]	Hold	75.0%	Presence of material in the pharynx ^b^	60.9%
Posterior spillage [Other than liquids]	Hold	75.0%	I. Volumes of material aggregating in the pharynx immediately before the pharyngeal swallow ^b^	61.0%
			II. Location of material in the pharynx ^c^	58.6%
Saliva swallow (to command)			Presence of voluntary swallow	80.5%
Sensory testing	Single, light touching of scope against different parts of the pharynx and larynx (including the arytenoids) ^d^	75.0%	Presence of motor response	75.6%
*Silent aspiration*: *As per Aspiration*			*As per Aspiration*	--
Spontaneous saliva swallow			Spontaneous swallowing (without bolus) present within a period of four minutes	75.6%
Swallow reaction time			I. Latency between the bolus reaching the pharynx and onset of swallow ^e^	70.7%
			II. Location of bolus head at onset of swallow^c^	65.8%
Symmetry			The symmetry of anatomy and movement ^d^	73.1%
Tracheal residue ^f^ [*See Aspiration and Silent Aspiration*]			*See Aspiration and Silent Aspiration*	--
Velopharyngeal closure	Saliva swallowing or add a sip of water (1 mL)	77.0%	Contact of velum with lateral and posterior walls of the pharynx	85.3%
Vocal fold medialisation	Phonating ‘eeee’ (or any other vowel)	86.5%	Degree and symmetry of closure of vocal folds	80.5%
White out			Presence of white-out	75.6%

^a^ Agreement with scale per operationalisation. ^b^ Reliability of estimating volumes to be tested in a future study. ^c^ Detailed descriptors of landmarks/areas are to be provided in the rating manual. ^d^ Minimal rewording using participants’ feedback. ^e^ Minimal rewording using participants’ feedback round III. ^f^ Item covered by other items.

**Table 5:** Agreement with definitions for relevant items (prototype measure).

Item (Alphabetical Order)	Definition	% Agreement ^a^	Delphi Round ^a^
Aspiration	The bolus or a portion of the bolus passes the level of the true vocal folds.	N.A.	N.A.
Bolus holding (to command)	The patient voluntarily holds the bolus in the oral cavity, attempting to prevent any bolus from escaping posterior.	N.A.	N.A.
Clearing swallow (pharyngeal)	An additional swallow initiated after the original bolus swallow is completed, triggered spontaneously by the patient in response to residue in the pharynx. No additional bolus material is added from the oral cavity during clearing swallows.	N.A.	N.A.
Cough (reflexive)	An involuntary cough is triggered when bolus enters the laryngeal vestibule.	N.A.	N.A.
Cough (voluntary)	The patient coughs in response to the clinician’s instruction.	N.A.	N.A.
Epiglottic retroflexion	Passive displacement of the epiglottis into the lumen of the larynx during swallowing.	N.A.	N.A.
Esophageal backflow	Return of material from the esophagus back into the pharynx.	97.2	III
Penetration	A portion of the bolus enters the laryngeal vestibule but does not pass below the vocal folds.	N.A.	N.A.
Pharyngeal constriction	Medial squeeze of the pharyngeal walls	88.0	I
Pharyngeal residue	After the total swallow is completed, bolus residue present in the pharynx.	N.A.	N.A.
Piecemeal deglutition	An additional swallow is initiated, and more bolus appears in the pharynx.	77.2	I
Pooling of secretions	Any material excluding bolus (e.g., mucus, saliva) visible in pharyngeal cavities or within the laryngeal vestibule at rest without/before testing with bolus.	80.5	III
Posterior spillage [Liquids]	Leakage of part of a liquid bolus into the pharynx during oral preparation, or before swallow initiation.	92.3	II
Posterior spillage [Other than liquids]	Leakage of a portion of a bolus into the pharynx during oral preparation or before swallow initiation.	78.9	II
Saliva swallow (to command)	Saliva swallowing upon request.	N.A.	II ^b^
Sensory testing	Testing sensation in the pharynx and larynx by light touching the scope against different parts of the pharynx and larynx in the craniocaudal direction, observing physical responses such as glottal closure.	76.4	I
Silent aspiration	The bolus or a portion of the bolus passes the level of true vocal folds and without causing a reflexive cough or throat-clearing response.	N.A.	N.A.
Spontaneous saliva swallow	Saliva swallowing is not upon request.	N.A.	II ^b^
Swallow reaction time	The time the bolus is in the pharynx until the swallow is triggered.	78.5	I
Symmetry	The symmetry of anatomy and movement (kinaesthetic aspects) of anatomical structures	82.3	III
Tracheal residue	After the pharyngeal swallow has been completed, bolus is present below the true vocal folds.	N.A.	N.A.
Velopharyngeal closure	The velum and lateral pharyngeal walls contractions, closing off the nasal cavity and nasopharynx.	89.3	I
Vocal fold medialisation	Moving the vocal folds towards the midline of the glottis	87.8	III ^b^
White out	A flash of intense white glare at the maximal constriction of the swallow due to the decreased distance between pharyngeal tissue and the light source.	79.7	I

^a^ N.A. (i.e., Not Applicable), agreement on items and definitions determined in Swan et al. (2021) [21]. ^b^ Original source Swan et al. (2021) [21]. Revision based on participants’ feedback Round II.

## Data Availability

Data is available on request due to ethical restrictions.

## Supplementary Materials

The following supporting information can be downloaded at: https://www.mdpi.com/article/10.3390/jcm12123875/s1, Supplementary File S1. Structure and content of Delphi rounds.
