# Supplementary material for: Development of a Visuoperceptual Measure for Fiberoptic Endoscopic Evaluation of Swallowing (V-FEES) in Adults with Oropharyngeal Dysphagia: An International Delphi Study
Source: J Clin Med. 2023 Jun 6;12(12):3875. doi: 10.3390/jcm12123875 (PMC10299684; doi:10.3390/jcm12123875)
Supplement: Supplementary file 1 [file jcm-12-03875-s001.zip › jcm-2404441-supplementary.pdf]

Development of a Visuoperceptual measure for Fiberoptic Endoscopic Evaluation of Swallowing (V-FEES) in adults with oropharyngeal dysphagia: an international Delphi study

Supplementary file S1. Structure and content of Delphi rounds.

ROUND I

- *Eligibility criteria* (fluency in English, working with adults with dysphagia *and* FEES);
- Questions on *Demographics* (profession, sector, qualification, primary role, patient populations, qualification, years of experience, country);
- List of definitions of key *terminology* in psychometrics and instrument development.

PART I

- List of definitions of observable items used in videofluoroscopic studies of swallowing that achieved international expert consensus during a Delphi study by Swan et al. (2021);
  Questions on the relevance of each item for FEES analysis using 5-point ordinal scales and open text boxes for additional comments.

> ***EXAMPLE Question***
>
> ***Aspiration***
> The bolus or a portion of the bolus passes the level of the true vocal folds.
>
> *Question*. This item is important to include:
> - ☐ Strongly disagree
> - ☐ Disagree
> - ☐ Neither agree nor disagree
> - ☐ Agree
> - ☐ Strongly agree
>
> *[Open text box if 'Strongly disagree' or 'Disagree']*
> Please indicate why you think this item is not important to include.

PART II

- List of additional definitions of observable items used in FEES as retrieved from literature and group discussions between the authors;
  Questions on relevance, agreement with definitions, and comprehensibility (i.e., easy to understand) using 5-point ordinal scales and open text boxes for additional comments.

> ***EXAMPLE Question***
>
> ***Swallow reaction time***
> Duration of time the bolus is in the pharynx until the swallow is triggered.
>
> *Question*. This item is important to include:
> - ☐ Strongly disagree
> - ☐ Disagree
> - ☐ Neither agree nor disagree
> - ☐ Agree
> - ☐ Strongly agree

[Open text box if 'Strongly disagree' or 'Disagree']
Please indicate why you think this item is not important to include.

Rate your level of agreement with this definition

- ☐ Strongly disagree
- ☐ Disagree
- ☐ Neither agree nor disagree
- ☐ Agree
- ☐ Strongly agree

[Open text box if 'Strongly disagree' or 'Disagree']
Please indicate what changes you would make to the definition and provide reasoning and/or references where able.

This item is easy to understand

- ☐ Strongly disagree
- ☐ Disagree
- ☐ Neither agree nor disagree
- ☐ Agree
- ☐ Strongly agree

NB. An item is considered important (i.e., relevant) if: 1) The item adds significant information which is relevant to FEES analysis, such as assisting with the diagnosis of underlying pathology, planning rehabilitation or assessing safety; and 2) The item should be routinely incorporated into FEES analysis.

ROUND II

- *Summary of Round I results*;
- *Eligibility criteria* (fluency in English, working with adults with dysphagia *and* FEES);
- Questions about *Demographics* (profession, sector, qualification, primary role, patient populations, qualification, years of experience, country).

PART I

- Two substantively revised and renamed items from Round I;
  Questions on relevance, agreement with definition and comprehensibility as per ROUND I, PART II.

PART II

- Overview of all included items from Round I;
  Request (open text box) to identify any missing, observable items important to current FEES instrument development (i.e., *comprehensiveness*).

PART III

- List of items and how to assess each item or aspect of the item (i.e., operationalisation). Examples of possible response scales are provided, and where applicable, tasks are described that need to be performed by patients (i.e., function testing) while observing and rating the item.
  Questions on level of agreement with patient tasks (where applicable) and operationalisations using 5-point ordinal scales and open text boxes for additional comments.

EXAMPLE Question

Bolus holding (to command)
Definition: The patient holds the bolus in the oral cavity voluntarily, attempting to prevent any bolus escape posteriorly.

Function testing: Hold bolus.

Question. Rate your level of agreement with the patient task (function testing):

- ☐ Strongly disagree
- ☐ Disagree
- ☐ Neither agree nor disagree
- ☐ Agree
- ☐ Strongly agree

[Open text box if 'Strongly disagree' or 'Disagree']
Please indicate how the function testing task can be improved or replaced.

Operationalisation: Presence of material in pharynx (e.g., nil present / small amount / large amount)

Question. Rate your level of agreement with the operationalisation of this item:

- ☐ Strongly disagree
- ☐ Disagree
- ☐ Neither agree nor disagree
- ☐ Agree
- ☐ Strongly agree

[Open text box if 'Strongly disagree' or 'Disagree']
Please indicate how the operationalisation of the item can be improved or replaced.

- Open text box to add any other comments.

ROUND III

- *Summary of Round II results*;
- *Eligibility criteria* (fluency in English, working with adults with dysphagia *and* FEES);
- Questions about *Demographics* (profession, sector, qualification, primary role, patient populations, qualification, years of experience, country).

PART I

- Two substantively revised and/or renamed items;
  Questions on agreement with definition and comprehensibility as per ROUND I, PART II.

PART II

- Four new items and definitions as recommended by participants during Round II (Comprehensiveness);
  Questions on relevance, agreement with definition and comprehensibility as per ROUND I, PART II.

PART III

- List of items and how to assess each item (i.e. operationalisation). Where applicable, tasks are described that need to be performed by patients (i.e., function testing) while observing and rating the item.
  Questions on level of agreement with operationalisations using 5-point ordinal scales and open text boxes for additional comments.

EXAMPLE Question

Posterior spillage [Other than liquids]
Definition: Leakage of a portion of a bolus into the pharynx during oral preparation, or before swallow initiation.

Operationalisation I: Presence of material in pharynx

1. Nil material present in the pharynx.
2. < one third of bolus present in pharynx.
3. ≥ one third of bolus present in pharynx.

Question. Rate your level of agreement with the operationalisation I of this item.

- ☐ Strongly disagree
- ☐ Disagree
- ☐ Neither agree nor disagree
- ☐ Agree
- ☐ Strongly agree

[Open text box if 'Strongly disagree' or 'Disagree']
Please indicate how the operationalisation I of the item can be improved or replaced.

Operationalisation II: Location of material in the pharynx

1. Nil material present in the pharynx
2. Material present in the upper oropharynx
3. Material present in the valleculae
4. Material present in the upper hypopharynx
5. Material present in the pyriform sinus
6. Material spread out through the pharynx (i.e., material present in more than one location)

Question. Rate your level of agreement with the operationalisation II of this item.

- ☐ Strongly disagree
- ☐ Disagree
- ☐ Neither agree nor disagree
- ☐ Agree
- ☐ Strongly agree

[Open text box if 'Strongly disagree' or 'Disagree']
Please indicate how the operationalisation II of the item can be improved or replaced.

- Open text box to add any other comments.

Reference

Swan, K.; Cordier, R.; Brown, T.; Speyer, R. Swan K, Cordier R, Brown T, Speyer R. Visuoperceptual analysis of the videofluoroscopic study of swallowing: an international Delphi study. *Dysphagia* 2021, 36, 595–613.
